# Gait and Lower Limb Observation of Paediatrics (GALLOP): development of a consensus based paediatric podiatry and physiotherapy standardised recording proforma

**DOI:** 10.1186/s13047-016-0139-4

**Published:** 2016-03-03

**Authors:** Simone Cranage, Helen Banwell, Cylie M. Williams

**Affiliations:** Peninsula Health-Community Health, PO Box 52, Frankston, Victoria 3199 Australia; School of Health Sciences, University of South Australia, PO Box 2471, Adelaide, South Australia 5001 Australia; School of Physiotherapy, Monash University, PO Box 527, Frankston, Victoria 3199 Australia

**Keywords:** Paediatric, Assessment, Physiotherapy, Podiatry, Lower limb, Delphi

## Abstract

**Background:**

Paediatric gait and lower limb assessments are frequently undertaken in podiatry and physiotherapy clinical practice and this is a growing area of expertise within Australia. No concise paediatric standardised recording proforma exists to assist clinicians in clinical practice. The aim of this study was to develop a gait and lower limb standardised recording proforma guided by the literature and consensus, for assessment of the paediatric foot and lower limb in children aged 0–18 years.

**Method:**

Expert Australian podiatrists and physiotherapists were invited to participate in a three round Delphi survey panel using the online Qualtrics^©^ survey platform. The first round of the survey consisted of open-ended questions on paediatric gait and lower limb assessment developed from existing templates and a literature search of standardised lower limb assessment methods. Rounds two and three consisted of statements developed from the first round responses. Questions and statements were included in the final proforma if 70 % or more of the participants indicated consensus or agreement with the assessment method and if there was support within the literature for paediatric age-specific normative data with acceptable reliability of outcome measures.

**Results:**

There were 17 of the 21 (81 %) participants who completed three rounds of the survey. Consensus was achieved for 41 statements in Round one, 54 statements achieved agreement in two subsequent rounds. Participants agreed on 95 statements relating to birth history, developmental history, hip measurement, rotation of the lower limb, ankle range of motion, foot posture, balance and gait. Assessments with acceptable validity and reliability were included within the final Gait and Lower Limb Observation of Paediatrics (GALLOP) proforma.

**Conclusion:**

The GALLOP proforma is a consensus based, systematic and standardised way to collect information and outcome measures in paediatric lower limb assessment. This standardised recording proforma will assist professions to collect information in a standardised format based on best evidence assessment methods whilst aiding consistency in communication between health professionals.

**Electronic supplementary material:**

The online version of this article (doi:10.1186/s13047-016-0139-4) contains supplementary material, which is available to authorized users.

## Background

Children’s gait and lower limb conditions are a frequent cause of parental concern and regularly present to podiatrists and physiotherapists [1]. Yet, very little is known on the consistency of the assessment methods conducted by podiatrists and physiotherapists specific to paediatric clients. Peninsula Health conducted a quality assurance review of assessment forms and recording proformas collected from a broad range of podiatry and physiotherapy paediatric services across the states and territories of Australia (Five University teaching clinics, five hospital outreach services and ten community health services). This review determined that the majority of services used a recording proforma to standardise data collection amongst clinicians; however, there was variation and inconsistency in the types of data collected, and the types of assessments conducted. Inconsistencies in data collection and assessment may lead to confusion between clinicians, increase repetition of measures and limits common language between assessment outcomes for both clinicians and clients alike. Furthermore, within the past 10 years, there have been a number of standardised assessments developed with paediatric age-specific normative values for the lower limb. The use of standardised and normative based assessments are important to use. To the best of our knowledge, however, no amalgamated proforma has been developed inclusive of these standardised assessments.

Paediatric specific outcome measures that evaluate the effectiveness of treatment within this population are becoming increasingly more important [1], they are also becoming more readily available. The Foot Posture Index-6 (FPI-6) [2], weight bearing lunge test [3], Beighton Scale and Lower Limb Assessment Scale [1, 4] have all demonstrated acceptable reliability within the paediatric population. Paediatric age-specific normative reference values for lower limb joint range, bone torsion and alignment have also been well documented [5–7] and these values are essential knowledge for the clinician in identifying deviation from typical development and to guide intervention requirements [5]. A standardised recording proforma offers many benefits. It directs clinicians on what to measure, and how to thoroughly record these outcome measures appropriately. It also ensures that the approach to foot or lower limb assessment undertaken across a broad range of services is based on best-available evidence, and standardises the information collected. This practice may also assist in the evaluation of variation from normal development and allows consistency in the monitoring of treatment outcomes specific to joint range of motion and gross motor development.

In the absence of existing foot or lower limb assessment guidelines, seeking expert podiatry and physiotherapy opinion through consensus gathering, is an appropriate first step to developing recommendations [8, 9]. The Delphi survey technique is a valid method to determine consensus, involving sequential questionnaires answered anonymously by a panel of participants with relevant expertise to gain consensus [9].

In summary, the aim of this research was to develop a gait and lower limb standardised recording proforma by expert consensus, which is supported by current literature for the assessment of paediatric gait and lower limb measures.

## Methods

### Design

The study was a four-round modified Delphi panel design. A modified Delphi survey was used where participants’ opinion was sought in round one, and then responses collated and analysed for existing consensus [10]. Responses not reaching consensus were returned to participants for consideration, comment and ranking for levels of agreement in subsequent rounds. The study was approved by the Peninsula Health Ethics Committee (LRR13PH24).

### Participants

Australian podiatrists and physiotherapists were invited to participate in the Delphi survey panel. For the purpose of this study, potential participants were identified based on their national reputation, extensive experience in paediatric assessment or by employment in a full time public health paediatric assessment role. Participants were also sought based on their authorship of literature appearing in paediatric specific journal publications, or where they held a teaching role in undergraduate paediatric specific subjects at an Australian University. Further potential participants were also identified from a review of the abstracts of national and state podiatry and physiotherapy conferences, and through contact of other clinicians within each field. Participants were considered enrolled once they supplied written informed consent. Intra-panel communication was anonymous and participants were asked to keep their involvement confidential.

### Procedure

The gait and lower limb assessment recording proforma from Australian universities, children’s hospitals and community health services with identified paediatric physiotherapy or podiatry services were reviewed where available. The individual assessment outcomes (including question based outcomes), related to the paediatric gait or lower limb, of each proforma were identified and collated.

A literature search was simultaneously conducted to determine the individual gait or lower limb assessment techniques reviewed within available evidence. The literature search used the PubMed database, based on the following search terms: “lower limb” OR “foot” OR “feet” OR “leg” OR “hip” AND “paediatric” OR “pediatric OR “child*” AND “assess*”. No quality assessment was applied to the results to ensure a broad representation of the literature was identified. An excel spreadsheet was used to document all the types of lower limb assessments that had been referenced within publications. The reliability and paediatric specific norms available on the assessment techniques that reached consensus or agreement within the Delphi survey were extracted.

The first round (round one) of the survey was developed based on the common items identified from the review of existing proformas and the literature. Round one was piloted by two podiatrists working in the paediatric field, with feedback supplied on language and usability of the questions. These podiatrists did not subsequently participate in the study.

Rounds two and three were based on the statements generated by participants in the first round. The aggregate anonymous answers were provided to all panel members at each stage along with the individual participant’s answers. Participants were asked to contact the authors should they feel their answers were in conflict with the subsequent rounds.

The surveys were implemented using the online survey platform Qualtrics^©^ [11]. Participants were requested to consider each question or assessment in relationship to the initial presentation of a child between the ages to 0–18. The open ended responses were collated into statements at the end of round one and complied for consensus by the authors (SC, CMW) with recommendations from the third author (HB) if conflict arose. Statements were considered to have reached consensus within Round one when 70 % or more of the participants indicated the same statement content by agreement of all three authors. This percentage is consistent with existing literature [12, 13]. Statements generated from round one were included in subsequent rounds and participants were asked to indicate their agreement with each statement on a five point Likert scale where 1 was Strongly Disagree, 2 was Disagree, 3 was Neutral, 4 was Agree and 5 was Strongly Agree. In the subsequent rounds, statements were considered accepted if 70 % or more of participants indicated that they agreed or strongly agreed with the statement. Statements not reaching 50 % of participant agreement were excluded. Statements receiving 50–69 % agreement were reviewed in subsequent survey rounds, when available, to ensure adequate panel consideration. Statements were excluded if agreement had not been achieved within two rounds. The a priori decision was that the Delphi would be concluded when the response rate dropped below 70 % or when round four was completed, irrespective of agreement. Participants were given 2 weeks to respond to each round, with a further 2 weeks offered where required. Feedback to participants and each subsequent round occurred within 4 weeks of the previous round.

### Analysis

Descriptive statistics were undertaken in Microsoft Excel 2010 (Microsoft Corp, Redmond Washington).

The responses were collated into the following sections: History, milestones, biomechanical, neurological and gait assessment. Questions resulting in a qualitative response (e.g. asking a parent to recall the timeline of milestones) were included if accepted (by consensus or agreement). The physical assessments that reached consensus or agreement with binary or categorical responses (e.g. the patella reflex defined as absent, typical or brisk) or continuous data output, were subject to review of acceptable reliability or paediatric specific norms prior to inclusion in the recording proforma. There are many aspects to the assessment of psychometric properties including internal consistency, validity, reliability and responsiveness [14]. Due to the volume of assessments generated throughout this process and the primary aim of the recording proforma, the authors only focused on the reliability of each assessment. This focus was aimed at ensuring the recording proforma was applicable to working environments where multiple clinicians may be taking the measurement. Accordingly, the reliability of the assessment was the only psychometric property considered. Studies reporting the reliability of each assessment were identified from a search of PubMed with the search terms “p$ediatric” AND “name of assessment” AND “reliability”. No formal quality assessment was made of each study but consideration was made of sample size, administration intervals, analysis of data and population group in relation to the context of this current study. Assessments were deemed appropriate where Intra class correlations (ICC) or Kappa agreement were greater than 0.61 (good agreement) [14]. Where more than one measure for the same assessment was agreed on by the panel, the assessment with the higher reliability was included. Where reliability of an assessment method was unable to be established, and no alternative (appropriate) paediatric specific assessment could be identified, a pragmatic approach was taken where the authors considered inclusion based on personal clinical experience.

## Results

Fifty potential participants were identified and invited to participate. Of the 21 participants who supplied written informed consent, 17 completed the initial questionnaire (81 %), (Table 1). Four participants were excluded as non-responders within the time period allotted. Of the 17 participants who participated in Round one, all 17 completed the subsequent rounds. Participants estimated that 68 % of their average caseload (clinical or research) was paediatric focused. There were no disagreements with participants on handling of their responses during any of the rounds.

**Table 1:** Participant Demographics

	Number (%)
Discipline	
Podiatry	12 (71)
Physiotherapy	5 (29)
Gender	
Male	5 (29)
Female	12 (71)
Years of Practice	
0–2 years	1 (6)
3–5 years	4 (24)
6–10 years	4 (24)
11–15 years	3 (18)
> 15 years	5 (29)
Original Qualification	
Diploma	1 (6)
Bachelor degree	15 (88)
Bachelor degree (Honours)	1 (6)
Further study completed in clinical discipline	
Graduate Certificate or Diploma	3 (33)
Masters by Coursework	4 (44)
Masters by Research	1 (11)
Professional Doctorate or Doctorate by Research (PhD)	2 (22)
Hours per week worked in primary job role	
0–10 hours	1 (6)
11–20 hours	2 (12)
21–30 hours	0 (0)
31–40 hours	9 (53)
41+ hours	5 (29)
Location of primary practice	
New South Wales	2 (12)
Queensland	1 (6)
South Australia	1 (6)
Victoria	11 (65)
Western Australia	2 (12)

### Consensus

Round one collected specific participant demographics and included open-ended questions of assessments previously identified in the literature search and the reviewed assessment forms (Additional file 1). Participants were asked to comment on each question with consideration of assessments used within their current practice. Comments were collated, with common themes identified and paraphrased into statements by discussion and agreement from the authors. The resulting statements were returned to the participants within Round two of the survey. Each participant also received their original individual responses from previous rounds to ensure satisfaction with the management of their comments. Round one generated 152 statements, of which, 41 statements reached consensus.

### Agreement

**Fig. 1:**
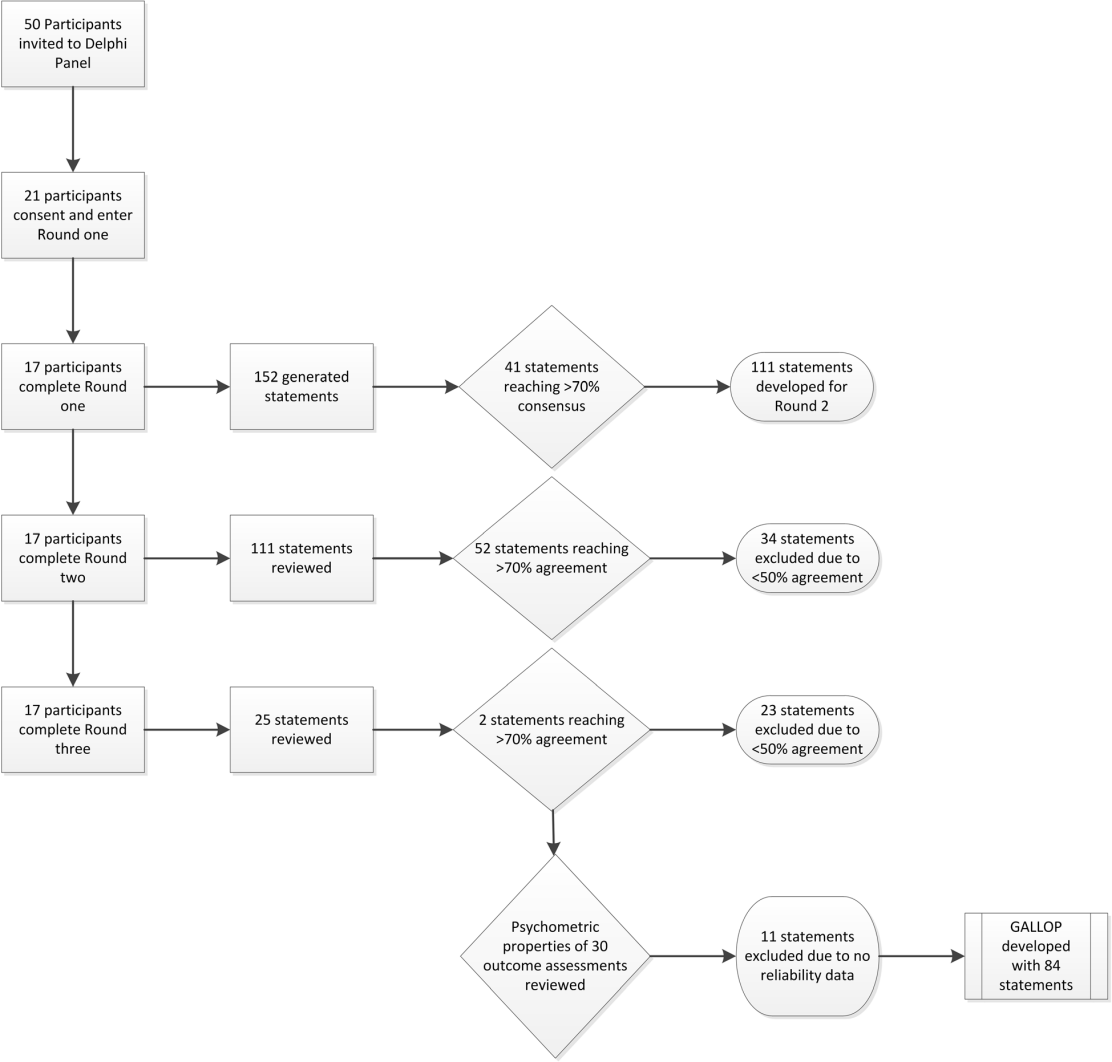
Consensus and agreement flow with number of statements accepted into the GALLOP

Round two (Additional file 2), participants reviewed 111 statements (Fig. 1). Of these 111 statements, 52 achieved >70 % agreement by participants, 25 statements achieved 50–69 % agreement and 34 statements were excluded (Fig. 1).

In Round three (Additional file 3), participants reviewed 25 statements (Fig. 1). Of these 25 statements, 2 statements achieved >70 % agreement and 23 statements were excluded (Fig. 1). Statements that reached the inclusion criteria for qualitative data are presented in Table 2.

**Table 2:** Qualitative history taking generated statements and assessments included within the GALLOP and round in which they were accepted

Question Theme	Generated statement	Consensus %	Agreement %	Round accepted
What are the common questions asked relating to pre and post natal history?	1. Pregnancy complications (Including mother’s health and medications)	88		1
	2. Term/gestation	100		1
	3. Delivery method (vaginal, spontaneous, induced caesarean, elective caesarean)	76		1
	4. Complications during and/or post-delivery and/or postnatal	100		1
	5. Breech position		94	2
	6. Birth weight		88	2
	7. Assistance during labour (Forceps/ventouse)		76	2
	8. APGAR score at 1 and 5 min		71	2
	9. Other health professionals involved at birth or in first 14 days		71	2
	10. Family history of foot or leg problems		100	2
Questions asked about the child’s developmental milestones or acquisition of skills	11. Age of sitting	71		1
	12. Age of crawling and type of crawl	100		1
	13. Age of walking	100		1
	14. Age of running	71		1
	16. Age of jumping		82	2
	17. Medical history/other health conditions		100	2
	18. Previous treatment of foot/lower limb		100	2
	19. Pain History		100	2
	20. Footwear		88	2
	21. Recreational/sporting history		88	2
	22. Sensory concerns		82	2
	23. Primary sitting positions		76	2
	24/25. Height/weight		71	3
Observation of presence in relationship to age	26. Squatting (single or double leg)	82		1
	27. Running	82		1
	28. Jumping	82		1
	29. Skipping	82		1
	30. Hopping	82		1
	31. Single leg stance- (eyes open, eyes closed, timed)	76		1
	32. Ability to go up/down stairs		76	2
	33. Observation of functional tasks (throwing, catching, kicking a ball, animal walks, sport specific activities)		71	2
	34. Quality of body movement (symmetry, coordination)		71	2
What other neurological observations should be recorded?	35. Muscle tone (passive, active, spasticity, rigidity)		88	2
	36. Gower’s sign		88	2
	37–39. Other observations (syndactlyl, skin folds of feet, legs or thighs, tufts)		76	2
What aspect of the lower limb are visualised during a gait assessment?	40. Arm swing (symmetry, guard position, flapping/flailing)	70		1
	41–43. Hip (rotation, flexion, hip drop/raise)	76		1
	44–50. Knee position (patella, flexion/extension/hyperextension, internal, frontal, external, genu varum/valgum, Q angle)	94		1
	51–55. Heel contact (initial contact, motion, timing, heel lift, rear-foot position)	82		1
	55. Mid-stance (mid-foot position)	76		1
	56–58. Toe off (Forefoot position during propulsion, symmetry, duration)	88		1
	59–61. Other gait observations (Trendelenberg, limp, circumduction or abductory twist etc.)	76		1
	62. Head and neck position	70		1
	63. Trunk/torso position and/or alignment		71	2
	64. Angle of gait (foot progression angle)		94	2
	65. Base of gait		82	2

### Development of results into a consensus based assessment proforma

The reliability of each physical assessment with continuous, binary or categorical outcome measures were collated and assessed against the inclusion criteria. Table 3 displays all of the assessments that were accepted by consensus or agreement by the Delphi panel and subsequent inclusion or exclusion based on acceptable reliability. The Gait and Lower Limb Observation of Paediatrics standardised recording proforma or GALLOP, was then developed from reliable, repeatable qualitative, categorical and continuous outcome measures. The proforma was collated and divided into a mix of questions aimed at data gathering and measures to aid differential diagnosis (Additional file 4).

**Table 3:** Physical assessment generated statements, round in which they were accepted, presence of paediatric ranges and if they were incorporated based on acceptable reliability

Question theme	Generated statements	Consensus %	Agreement %	Round Accepted	Paediatric ranges or values	Incorporated into final proforma
What reflexes should be tested in the lower limb?	66. Patella (knee jerk, quadriceps) (Grading 0–4)	94		1	N/A	Yes [25]
	67. Achilles (ankle jerk, gastrocnemius) (Grading 0–4)	84		1	N/A	Yes [25]
	68. Plantar reflex (up or down going)	71		1	N/A	Yes [26]
What other neurological observations should be recorded?	69. Presence of ankle catch (R1/R2)		88	2	N/A	Yes [25]
	70. Presence of ankle clonus		94	2	N/A	Yes [25]
	71. Dorsiflexion strength (Grading 0–5)		71	2	No	Yes [27]
	72. Plantarflexion strength (Grading 0–5)		71	2	No	Yes [27]
	73. Inversion strength (Grading 0–5)		71	2	No	Yes [27]
	74. Eversion strength (Grading 0–5)		71	2	No	Yes [27]
	75. Beighton score		94	2	Yes [28]	Yes [28]
How is hip range of movement measured?	76. Internal/external rotation with the knee flexed and extended)	76		1	Yes [3, 5]	Yes [3]
	77. Modified Thomas test	71		1	Yes [5, 29]	Yes [30]
	78. Hip abduction	71	8	1	Yes [5, 31]	Yes [31]
How is hamstring range of movement measured?	79. Popliteal angle	88		1	Yes [7]	Yes [5]
How leg length measured?	80. Observation (frontal plane pelvic/shoulder tilt, scoliosis check, knee creases, head tilt, foot posture, gait)		94	2	N/A	Yes [24]
How is the rotational profile measured?	81. Foot thigh angle		82	2	Yes [5]	Yes [32]
	82. Other observations:(metatarsus adductus)	76	82	2	Yes [33]	Yes^a^
How is ankle range of movement measured?	83. Non weight bearing ankle dorsiflexion with knee extended and knee flexed)	94		1	Yes [5]	Yes^b^[34]
	84. Weight bearing lunge test (knee extended and flexed)	76	82	2	Yes [3, 35]	Yes [1]
	85. Observed ability to squat	88	88	2	No	No
	84. Observed ability to walk on toes	88	88	2	No	No
	85. Observed ability to heel walk	88	88	2	No	No
How is the presence of a genu valgum/genu varum measured?	86. Inter-condylar/inter-malleolar distance (Weight-bearing)	82		1	Yes [36]	Yes [37]
How is foot posture measured?	87. Foot Posture Index-6	76		1	Yes [2]	Yes [38]
	88. Hubscher maneuver (Jack’s test)		82	2	No	No
	89. Subtalar joint axis		71	2	No	No
	90. Subtalar joint ROM		71	2	No	No
	91. Forefoot to rearfoot relationship		71	2	No	No
	92. Midtarsal joint non weightbearing ROM		71	2	No	No
	93. 1st MPJ Non weightbearing ROM		76	2	No	No
	94. Ability to stand on tip toes		82	2	No	No
	95. Does the rearfoot resupinate while on tiptoes		82	2	No	No

^a^No reliability results available

^b^Reliability lower than weightbearing lunge however recommended when a child is unable to place their heels on the ground

## Discussion

This study has resulted in the first consensus-based standardised recording proforma for collation of history and lower limb and foot assessment measures designed for use in the paediatric population by podiatrists and physiotherapists. This study has used face validity to provide clinician agreement on which common assessments should be considered for use within the clinical setting. Clinicians, researchers and students can consider this proforma a useful amalgamated resource of contemporary assessments to guide clinical practice.

Birth and developmental history questions are an important component of paediatric history taking to identify potential concerns or raise red flags for disease processes. For example, infants who are born preterm or secondary to intrauterine growth restriction often present with gait or gross motor concerns [15]. Low birth weight has been associated with cognitive delay, cerebral palsy and can be associated with a greater risk of chronic medical conditions in later life [16]. Parent recall of birth and developmental history has been reported as adequate [17–19] therefore it is relevant that these questions be an important component of the GALLOP. Categorical and quantitative outcome measures are also essential in diagnosis and for evaluation of treatment in paediatric populations. For example, the use of the FPI-6 as an established reliable and valid measure of foot posture allows the clinicians to measure any change over time with growth or disease progression [20]. The measures involved in gait assessment proved to be challenging, with visual assessment and quantitative recorded observations being the preferred method. This was also the area with limited reliability data. Accordingly, the recommendation is that clinicians should view visual gait analysis with caution.

The GALLOP is an aggregation of assessments, it is expected that some questions and assessments within this proforma may not be appropriate for every paediatric client. While it is a comprehensive collection of questions and measures, it is recommended that clinicians use their clinical judgement and expertise in its use. Clinicians should also be well versed in understanding when other screening tools may be required, or when items can be omitted based on presentation and age of the paediatric client. For example, the child presenting with pain in multiple joints may require the use of the Paediatric Gait Arms and Legs (pGALS) tool [21] or the child presenting with toe walking, should trigger the use of the Toe Walking Tool [22]. Similarly, the use of standardised gross motor assessment tools, such as the Bruininks-Oseretsky Test of Motor Proficiency-2 [23], may be required for indepth analysis of the child who is unable to demonstrate age appropriate gross motor skills.

It is also important to acknowledge a number of limitations within this study. There were a number of components included within the proforma that do not have acceptable reliability data, specifically those related to visual gait analysis, leg length discrepancy and metatarsus adductus. As the aim of the GALLOP was to assist clinicians in all settings, it was proposed that guiding the clinician in these assessments was preferable to removing these accepted aspects from the final proforma. With time and increased feasibility of gait analysis equipment, technology that increases the reliability of findings may be more frequently available in practices and replace the current qualitative gait descriptors. Furthermore, the use of reliable and accurate clinical and imaging modalities for quantifying of orthopaedic concerns (e.g. leg length discrepancy or metatarsus adductus) is essential when planning for appropriate treatment. It is considered that the assessment methods retained within the GALLOP, such as use of a tape measure and standing blocks may be useful as a screening tool [24], but caution should be applied to their interpretation. Assessments without acceptable or any reliability values are indicated by an asterisk within the GALLOP.

The GALLOP was based on expert opinion, which in the context of evidence based practice constitutes low level evidence. Author bias may have been introduced during reduction of assessments included within the final model of the proforma, however, the authors have attempted to minimise this with transparency of the implemented process. The Delphi survey process has also been subject to concerns, with the existence of consensus or agreement not ensuring correctness [9]. Furthermore, remaining anonymous and confidential are suggested requirements of participants in Delphi surveys to minimise the effects, if any, of collusion on results. Given the collegiate relationships that exist within the Australian podiatry and physiotherapy professions, it cannot be guaranteed that participants remained anonymous to their colleagues. All participants were cautioned to keep both their responses and participation confidential to minimise this risk.

The term “expert” and its application to health professionals is controversial, as no classification exists within the podiatry profession and the term is limited within the physiotherapy profession for expert status within the area of paediatrics. The criteria set for expert within this study were based on previous criteria within Delphi panels to ensure that participants had recognised expertise within their respective professions [12].

It is also recognised that gait and lower limb conditions present to a wider range of health professionals. The aim of this study was to develop a recording proforma used for generalised initial paediatric lower limb assessment and gait for allied health clinicians, therefore this proforma may be limited to only physiotherapists and podiatrists use. Locality of participants may also be a limitation as all practice within Australia, however, international research used for the inclusion criteria may have minimised this impact. Lastly, as the GALLOP was developed as an aggregation of parent reported items and validated outcome measures, it is unable to provide a final quantitative score. Rather, it acts as an aid to record outcomes and assists in clinical decision making in relation to diagnosis and treatment recommendations for foot and leg conditions in the paediatric client. It is possible that other assessments, with lower reliability, may be appropriate for the clinician to employ depending on the ability of the child or presenting complaint (i.e. non-weight bearing ankle range of motion with leg straight and knee bent). Many factors impact on the reliability of an assessment, these include the population group, age group, clinician experience or measurement apparatus. It is important for clinicians to be aware of these implications when undertaking each assessment. This proforma should not be used in place of good clinical judgement or where there is evidence to support additional or different assessments or measurements.

There are numerous benefits to professions and clients using a single data collection form. Collection of uniform data across a country or even internationally is of interest to researchers seeking to determine longitudinal change or lower limb associative factors of disease process. As many undergraduate podiatry courses have student led clinics, embedding the GALLOP into teaching, offers consistency for the next generation of clinicians and may also provide collaborative opportunities for research projects across universities. The use of this proforma may also stimulate future research into the sensitivity and validity of assessments contained within the GALLOP as they have been identified by experts as important clinical measures.

## Conclusion

The GALLOP should be considered by physiotherapists and podiatrists as a systematic method of collecting information and outcome measures relating to the foot and lower limb. The use of a universal proforma will assist clinicians to collect information in a similar format therefore aiding communication between professions and to other health professionals.

## Additional files

Additional file 1: GALLOP- Round 1. (PDF 279 kb) Additional file 2: GALLOP- Round 2. (PDF 272 kb) Additional file 3: GALLOP- Round 3. (PDF 251 kb) Additional file 4: GALLOP recording proforma. (DOCX 118 kb)
