# Supplementary material for: Gait and Lower Limb Observation of Paediatrics (GALLOP): development of a consensus based paediatric podiatry and physiotherapy standardised recording proforma
Source: J Foot Ankle Res. 2016 Mar 3;9:8. doi: 10.1186/s13047-016-0139-4 (PMC4778335; doi:10.1186/s13047-016-0139-4)
Supplement: Additional file 1: — GALLOP- Round 1. (PDF 279 kb) [file 13047_2016_139_MOESM1_ESM.pdf]

Supplementary File 1 - GALLOP – Round one.

Thank you for participating in this research. You have been invited to take part in this research project as you have been identified as having experience in the field of paediatric assessment in the podiatry and physiotherapy profession. This project aims to develop a lower limb assessment tool that can be used Australia wide to gain consistency in the type of questioning and assessment that is used in young children. Please enter your participant number. You can go back to any question as required. Your responses are confidential and will only be known by the research team. The survey should take 20 minutes to complete. If you have any questions please contact Simone Cranage (scranage@phcn.vic,gov.au) Cylie Williams (cyliewilliams@phcn.vic.gov.au) Helen Banwell (HelenBanwell@unisa.edu.au)

Thank you for participating in this research.

This project aims to develop a lower limb assessment tool that can be used to gain consistency in the type of questioning and assessment that is used with children.

To assist you in your responses, it may help if you have a copy of any assessment form that you currently use when assessing children.

Your responses are confidential and will only be known by the research team.

The survey should take 20 minutes to complete.

If you have any questions please contact Simone Cranage (scranage@phcn.vic,gov.au) Cylie Williams (cyliewilliams@phcn.vic.gov.au) or Helen Banwell (HelenBanwell@unisa.edu.au)

Q1. What is your participant number?

Q2. Gender

- Male
- Female

Q3. What is your profession?

- Podiatrist
- Physiotherapist

Q4. How many years have you been practicing (in full time equivalence)?

- 0-2 years
- 3-5 years
- 6-10 years
- 11-15 years
- >15 years

Q5. What percentage do you practice in the following settings? (Please enter number value)

Private sector- Private practice	0
Public sector- Community Health service	0
Public sector- hospital	0
Non-clinical (Includes: Administrative and/research positions)	0
**Total**	0

Q6. What was your original qualification?

- Certificate
- Advanced Certificate
- Bachelor Degree
- Bachelor Degree with Honors
- Masters (entry level)
- Other (Please Specify)

Q7. Where did you obtain your original qualification?

Q8. Have you completed further tertiary study?

- Yes
- No

Q9. Please indicate which further study you have completed in your clinical discipline (You may choose more than one option)

- Graduate Certificate
- Graduate Diploma
- Masters by Coursework
- Masters by Research
- Professional Doctorate
- Doctorate by Research (PhD)
- Other (Please specify)

Q10. Are you currently undertaking further study in your clinical discipline?

- Yes
- No

Q11. Please indicate which further study you are currently undertaking (You may choose more than one option)

- Graduate Certificate
- Graduate Diploma
- Masters by Coursework
- Masters by Research
- Professional Doctorate
- Doctorate by Research (PhD)
- Other (Please specify)

Q12. What year did you graduate with your highest academic qualifications?

Q13. On average how many hours per week do you work at your primary position?

- 0-10 hours
- 11-20 hours
- 21-30 hours
- 31-40 hours
- 41 + hours

Q14. Where do you primarily practice?

- Australian Capital Territory
- New South Wales
- Northern Territory

- Queensland
- South Australia
- Tasmania
- Victoria
- Western Australia

Q15. Please estimate the percentage of children you work with in your clinical or research role.

	0	20	40	60	80	100
% of children						

Q16. For the children you see in your practice, what is the most common age group? (You may select more than one option)

- 0 years -3.0 years
- 3.1 years -6.0years
- 6.1 years -10 years
- 10.1 years -18 years

For the following questions, we would ask you to consider your practice when taking history and assessing a child who has attended for a full foot and leg examination. We recognise that children can be difficult to assess at times. Please comment as though you had the "perfect" child that is compliant to assessment.

Q17. Please list the common questions you would ask relating to prenatal and postnatal history? (Prenatal includes pregnancy and post natal includes day of birth and subsequent hours)

Q18. Please list the questions you would ask about the child's acquisition of skills or developmental stages?

Q19. Please list any other questions you would ask during history taking that are not covered by the previous questions? (If there are no further questions please indicate N/A in response box)

Assessment

When assessing a child please comment on the following.

You may list the assessment if it has a name otherwise describe how the assessment is performed

Q20. What assessments do you perform with the child standing?

Q21. What assessments do you perform with the child seated?

Q22. What assessments do you perform with the child in supine? (lying on their back)

Q23. What assessments do you perform with the child in prone? (laying on their tummy)

Measurements
For the following measurements, please comment on the method, name of assessment, and the measurement tool you commonly use (if you use one)

If you have more than one, please comment on them all. ie. Hip measurement: Internal and external ROM with eyeball gestimate or tractograph.

If you do not measure any one item, please indicate N/A in response box.

Q24. How do you measure hip range of movement?

Q25. How do you measure hamstring range of movement?

Q26. How do you measure leg length?

Q27. How do you measure rotational profile of the upper and lower leg?

Q28. How do you measure ankle range of movement?

Q29. How do you measure the presence of genu varum/genu valgum?

Q30. How do you measure the foot posture?

Q31. How do you measure gross motor ability and/or balance?

Q32. What reflexes do you test in the lower limb?

Q33. What other aspects of a neurological assessment do you consider?

Q34. Are there any other measures that you routinely use?

Q35. Gait analysis
Please describe what aspects of the body and the lower limb you visualise during a gait assessment. (i.e shoulder level looking for symmetry etc)

Q36. How do you measure and document these?

Thank you for taking the time to complete this survey. The next round will be emailed to you as soon as possible.
By clicking out of this survey you will not be able to re-enter and your answers will be saved.

If you would like to modify your responses, please use the back button or close and re-enter at a later date. Please remember you only have 2 weeks from this time to complete the survey. Your responses though will not be recorded until you click the button at the end of this question.
