# Supplementary material for: Gait and Lower Limb Observation of Paediatrics (GALLOP): development of a consensus based paediatric podiatry and physiotherapy standardised recording proforma
Source: J Foot Ankle Res. 2016 Mar 3;9:8. doi: 10.1186/s13047-016-0139-4 (PMC4778335; doi:10.1186/s13047-016-0139-4)
Supplement: Additional file 2: — GALLOP- Round 2. (PDF 272 kb) [file 13047_2016_139_MOESM2_ESM.pdf]

Supplementary File 2 - GALLOP – Round two.

University of
South Australia

Thank you for participating in this research. You have been invited to take part in this research project as you have been identified as having experience in the field of paediatric assessment in the podiatry and physiotherapy profession. This project aims to develop a lower limb assessment tool that can be used Australia wide to gain consistency in the type of questioning and assessment that is used in young children. Please enter your participant number. You can go back to any question as required. Your responses are confidential and will only be known by the research team. The survey should take 20 minutes to complete. If you have any questions please contact Simone Cranage (scranage@phcn.vic,gov.au) Cylie Williams (cyliewilliams@phcn.vic.gov.au) Helen Banwell (HelenBanwell@unisa.edu.au)

Thank you for participating in this research.

This project aims to develop a lower limb assessment tool that can be used to gain consistency in the type of questioning and assessment that is used with children.

To assist you in your responses, we have also emailed you your responses from round 1.

Your responses are confidential and will only be known by the research team.

The survey should take under 15 minutes to complete. You will have the chance at the end to make any comments if you wish.

If you have any questions please contact Simone Cranage (scranage@phcn.vic,gov.au) Cylie Williams (cyliewilliams@phcn.vic.gov.au) or Helen Banwell (HelenBanwell@unisa.edu.au)

Q1. What is your participant number?

For the following questions, we would ask you to consider your practice and original responses when taking history and assessing a child who has attended for a full foot and leg examination.

We have collated the responses from all the participants thus far into themes and will report on consensus of these. Consensus was deemed as 70% agreement. For the items that consensus was not achieved, we ask you to respond with your agreement of whether the question or measure should be included.

1. Pre and post natal history

Based on the responses, consensus was achieved on the following themes (70% of people identified these themes).

Therefore the pre and post natal history questions that the parent/caregiver should be asked is:
1. Were there any complications during pregnancy? (Prompts: Health of the mother, use of medications) Yes No
List:____
2. What was the term of the pregnancy? _________wks?
3. How was the baby delivered Vaginal Caesarean
4. List any post-natal complications during or post delivery (Prompts: Resuscitations, NICU, Special Care Nursery, congential abnormalities, medications, general health).

The following themes were also identified, however consensus was not achieved. Please rate on the following scale your opinion of whether these statements/questions should be included:

	Strongly Disagree	Disagree	Neutral	Agree	Strongly Agree
Labour	○	○	○	○	○
Breech	○	○	○	○	○
Birth weight	○	○	○	○	○
Assistance required during labour (forceps/ventouse)	○	○	○	○	○
APGAR Score	○	○	○	○	○
Other medical professionals involved in the care of the baby at birth or within the first 14 days?	○	○	○	○	○
Any prior pregnancies?	○	○	○	○	○
Immunisation	○	○	○	○	○

Family history of foot or leg problems	○	○	○	○	○

2. Developmental milestones/history

Based on the responses, consensus was achieved on the following themes (70% of people identified these themes).
Therefore the proposed developmental milestones/history questions that the parent/caregiver should be asked is:

Age of Sitting ______months
Age of Crawling ________________________months Type of crawl_________________________
Age of Walking _____________months Running _______________________years

The following themes were also identified however consensus was not achieved. Please rate on the following scale your opinion of whether these statements/questions should be included:

	Strongly Disagree	Disagree	Neutral	Agree	Strongly Agree
Age of pull to stand	○	○	○	○	○
Begin to squat	○	○	○	○	○
Age of beginning to cruise	○	○	○	○	○
Age of beginning to jump	○	○	○	○	○
Age of beginning to hop	○	○	○	○	○
Age of beginning to skip	○	○	○	○	○
Age of walking up/down stairs	○	○	○	○	○
Age of beginning to kick a ball	○	○	○	○	○

3. Other history questions

Based on the responses regarding other questions asked during history taking, no consensus was achieved; however the following themes were identified. Please rate on the following scale your opinion of whether these statements/questions should be included:

	Strongly Disagree	Disagree	Neutral	Agree	Strongly Agree
Medical history	○	○	○	○	○
Previous treatment of foot or lower limb (interventions, orthotics, AFO's)	○	○	○	○	○
Pain History	○	○	○	○	○
Footwear	○	○	○	○	○

Recreational/Sport activities/Social history	◯	◯	◯	◯	◯
Sensory concerns (surfaces/textures)	◯	◯	◯	◯	◯
Parental goals of treatment	◯	◯	◯	◯	◯
Primary sitting position	◯	◯	◯	◯	◯
Primary sleeping position	◯	◯	◯	◯	◯
Height/weight	◯	◯	◯	◯	◯

4. Hip Assessment

Based on the responses, consensus was achieved on the following themes (70% of people identified the following assessments used when assessing hip range of movement and the preferred measurement method). Therefore the following hip assessments to be incorporated into the GALLOP assessment are:

Internal/external rotation (knee flexed and/or extended) Left__________ Right____________

Thomas test (modified thomas test) Left__________________ Right ____________________

Hip abduction Left__________________ Right ____________________

Hip adduction Left _________________ Right _____________________

Preferred measurement methods: Goniometer

The following assessments were also identified however consensus was not achieved. Please rate on the following scale your opinion of whether the following assessments should be included when measuring hip range of movement:

	Strongly Disagree	Disagree	Neutral	Agree	Strongly Agree
Quad ROM/tightness	◯	◯	◯	◯	◯

5. Hamstring Assessment

Based on the responses, consensus was achieved on the following themes (70% of people identified the following assessments used when assessing hip range of movement and the preferred measurement method). Therefore the following hip assessments to be incorporated into the GALLOP assessment are:

Popliteal angle: Left ________________ Right _________________

Method: Angle finder

The following assessments were also identified however consensus was not achieved. Please rate on the following scale your opinion of whether the following assessments should be included when measuring hip range of movement:

	Strongly Disagree	Disagree	Neutral	Agree	Strongly Agree
Straight leg raise	○	○	○	○	○
Functional- long legged sitting	○	○	○	○	○

6. Leg Length Assessment

Based on the responses, consensus was not achieved on measurement of a leg length difference. The following methods of assessment and tools were identified. Please rate on the following scale your opinion on the inclusion of the following methods to assess for a limb length discrepancy

	Strongly Disagree	Disagree	Neutral	Agree	Strongly Agree
ASIS-MM (Supine or standing)	○	○	○	○	○
Umb-MM (Supine or standing)	○	○	○	○	○
ASIS-plantar surface of heel/ground	○	○	○	○	○
ASIS-plantar surface of heel/ground	○	○	○	○	○
Umb-plantar surface of heel/ground	○	○	○	○	○
ASIS-head of fibula	○	○	○	○	○
Comparison of ASIS and PSIS height	○	○	○	○	○
Stance umb-floor	○	○	○	○	○
Galleazi	○	○	○	○	○
Observation (frontal plane pelvic/shoulder tilt, scoliosis check, knee creases, head tilt, foot posture, gait)	○	○	○	○	○

7. Leg Length method of measurement

Based on the responses, consensus was not achieved on measurement tools used to assess of a leg length difference. The following methods of assessment and tools were identified. Please rate on the following scale your opinion on the inclusion of the following measurement tools

	Strongly Disagree	Disagree	Neutral	Agree	Strongly Agree

Palpation	○	○	○	○	○
Use of blocks	○	○	○	○	○
Imaging (X-ray, CT)	○	○	○	○	○
Eyeball	○	○	○	○	○
Tape measure	○	○	○	○	○

8. Rotational Profile

Based on the responses, consensus was achieved on the following themes (70% of people identified the following methods and tools when assessing rotational profile of the upper and lower leg. Therefore the following assessments to be incorporated into the GALLOP are:

Internal Hip ROM (hip flexed/extended, knee flexed/extended) Left___________ Right___________
External Hip ROM (hip flexed/extended, knee flexed/extended) Left ___________ Right ___________

The following assessments were also identified however consensus was not achieved. Please rate on the following scale your opinion of whether the following assessments should be included when measuring rotational profile of the upper and lower leg.

	Strongly Disagree	Disagree	Neutral	Agree	Strongly Agree
Ryder's Test	○	○	○	○	○
Genicular rotation	○	○	○	○	○
Thigh foot angle (tibial torsion)	○	○	○	○	○
Trans-malleolar angle/malleolar position	○	○	○	○	○
Other (lumbar spine curve, shoulder symmetry, ASIS, knee position, metatarsus adductus)	○	○	○	○	○

9. Rotational profile method of measurement.

Please rate on the following scale your opinion on the most appropriate tool when assessing the rotational profile of the upper and lower leg

	Strongly Disagree	Disagree	Neutral	Agree	Strongly Agree
Goniometer	○	○	○	○	○
Digital inclinometer	○	○	○	○	○

Smart phone app (tilt meter etc.)	○	○	○	○	○
Eyeball	○	○	○	○	○

10. Ankle range of movement

Based on the responses, consensus was achieved on the follow themes (70% of people identified the following assessments used when assessing ankle range of movement. Therefore the following assessments to be incorporated into the GALLOP are:

1. Non weight-bearing ankle dorsiflexion (knee extended) Left __________ Right ___________
2. Non weight-bearing ankle dorsiflexion (knee flexed) Left ____________ Right ____________

The following assessments were also identified however consensus was not achieved. Please rate on the following scale your agreement of whether the following assessments should be included when measuring ankle range of movement

	Strongly Disagree	Disagree	Neutral	Agree	Strongly Agree
Weight-bearing lunge test (knee flexed, knee extended)	○	○	○	○	○
Functional testing (ability to squat, walk on toes, walk on heels)	○	○	○	○	○

11. Ankle range of movement measurement

Please rate your level of agreement of which you believe should be used as the primary recommended tool when measuring ankle range of movement

	Strongly Disagree	Disagree	Neutral	Agree	Strongly Agree
Goniometer	○	○	○	○	○
Digital inclinomter	○	○	○	○	○
Eyeball measurement	○	○	○	○	○

12. Presence of a genu varum/genu valgum

Based on the responses, consensus was achieved on the following themes (70% of people identified the following assessments used measuring the presence of genu varum/genu valgum. Therefore the following assessments to be incorporated into the GALLOP are:

1. Intercondylar distance (WB) ________cm
2. Intermalleolar distance (WB) ________ cm
3. Use of a tape measure/ruler

The following assessments were also identified however consensus was not achieved. Please rate on the following scale your opinion of whether the following assessments should be included when measuring the presence of a genu varum/genu valgum

	Strongly Disagree	Disagree	Neutral	Agree	Strongly Agree
Intercondylar distance (non-WB)	○	○	○	○	○
Intermalleolar distance (non-WB)	○	○	○	○	○
Angle of ASIS/patella tendon/tibial spine	○	○	○	○	○
Axial tibial/fibula angle	○	○	○	○	○

13. Foot Posture

Based on the responses, consensus was achieved on the following themes (70% of people identified the following assessments used when assessing foot posture. Therefore the following assessments to be incorporated into the GALLOP are:

1. FPI-6 Left ___________ Right _____________

Method of measurement: Eyeball

The following assessments were also identified however consensus was not achieved. Please rate on the following scale your opinion of whether the following assessments should be included when assessing foot posture

	Strongly Disagree	Disagree	Neutral	Agree	Strongly Agree
Resting Calcaneal Stance Position	○	○	○	○	○
Neutral Calcaneal Stance Position	○	○	○	○	○
Navicular height/drop	○	○	○	○	○
Supination resistance	○	○	○	○	○
Presence of an arch (non-WB)	○	○	○	○	○
Jack's test	○	○	○	○	○
Sub talar joint axis/ROM	○	○	○	○	○
Mid-tarsal joint axis/ROM	○	○	○	○	○
1st MPJ ROM	○	○	○	○	○

1st metatarsal length	◯	◯	◯	◯	◯
Forefoot to rearfoot relationship	◯	◯	◯	◯	◯
Plantar facial prominence	◯	◯	◯	◯	◯
Standing on tip toes (STJ inversion, tibialis posterior function)	◯	◯	◯	◯	◯

14. Gross motor ability/balance

Based on the responses, consensus was achieved on the following themes (70% of people identified the following assessments used when measuring gross motor ability and/or balance. Therefore the following assessments to be incorporated into the GALLOP are:

Observations of achievement:

1. Squatting (single or double)
2. Running
3. Jumping
4. Skipping
5. Hopping
6. Single leg stance (eyes open, timed) Left ________ Right ____________
7. Single leg stance (eyes closed, timed) Left _________ Right ___________

The following assessments were also identified however consensus was not achieved. Please rate on the following scale your opinion of whether the following assessments should be included when INTIALLY measuring gross motor ability and/or balance.

	Strongly Disagree	Disagree	Neutral	Agree	Strongly Agree
Use of standardised assessment (Himat, BOT-2, Berg balance scale, AIMS, Peabody, ASQ etc.	◯	◯	◯	◯	◯
Heel to toe stationary (eyes open, eyes closed)	◯	◯	◯	◯	◯
Walking heel to toe along straight line (eyes open, eyes closed)	◯	◯	◯	◯	◯
Climbing ability	◯	◯	◯	◯	◯
Ability to go up/down stairs	◯	◯	◯	◯	◯
Balance beam	◯	◯	◯	◯	◯
Other observations (fatigue, agility, maturity of pattern, coordination of movements, symmetry, strength)	◯	◯	◯	◯	◯
Other functional tasks (throwing, catching, kicking a ball, animal walks, sport specific activities)	◯	◯	◯	◯	◯
Ability to walk on toes	◯	◯	◯	◯	◯
Ability to walk on heels	◯	◯	◯	◯	◯

Maximum number of heel raises	◯	◯	◯	◯	◯
Presence of a reciprocal arm swing	◯	◯	◯	◯	◯
6WT, 10RT	◯	◯	◯	◯	◯
Times supine to stand	◯	◯	◯	◯	◯
Quality of movement (symmetry, coordination)	◯	◯	◯	◯	◯

15. Reflexes

Based on the responses, consensus was achieved on the following themes (70% of people identified the following reflexes tested in the lower limb. Therefore the following reflexes to be incorporated into the GALLOP are:

1. Patella (knee jerk, quadriceps) reflex
2. Achilles (ankle jerk, gastrocnemius) reflex
3. Plantar reflex (up or down going)

The following reflex was also identified however consensus was not achieved. Please rate on the following scale your opinion of whether the following reflexes should be included in your assessment

	Strongly Disagree	Disagree	Neutral	Agree	Strongly Agree
Chaddock	◯	◯	◯	◯	◯

16. What is your preferred method of grading responses

◯ 0 = No response
1+ = slight by definitely present response
2+ = brisk response
3+ = very brisk response
4+ = brisk with clonus

◯ 0: Absent reflex
1+: trace, or seen only with reinforcement
2+: normal
3+: brisk
4+: nonsustained clonus (i.e., repetitive vibratory movements)
5+ sustained clonus

◯ Other

17. Neurological other

Based on the responses, no consensus was achieved when asked about what other aspects of a neurological assessment are considered. The following assessments were identified. Please rate on the following scale your opinion of which other aspects of a neurological assessment should be considered.

	Strongly Disagree	Disagree	Neutral	Agree	Strongly Agree
Muscle tone (passive, active, spasticity, rigidity)	○	○	○	○	○
Presence of a catch (R1/R2)	○	○	○	○	○
Presence of a clonus	○	○	○	○	○
Clasp knife	○	○	○	○	○
Sensory assessment (perception, discrimination, sensation, proprioception, monofilament)	○	○	○	○	○
Gower's sign	○	○	○	○	○
Hand to nose (eyes closed)	○	○	○	○	○
Muscle strength/muscle testing	○	○	○	○	○

18. Assessment other

Based on the responses, no consensus was achieved when considering other measures that are used routinely during an assessment. The following assessments were identified. Please rate on the following scale your opinion of which other measures or assessments should be routinely considered.

	Strongly Disagree	Disagree	Neutral	Agree	Strongly Agree
Beighton score	○	○	○	○	○
Knee tests (ligament, menisci, PFJ tests, ACL etc.)	○	○	○	○	○
Tibiofibular joint mobility	○	○	○	○	○
Toe walking tool	○	○	○	○	○
Other assessment (spinal examination, PAIVMs)	○	○	○	○	○

Other observations (syndactlyl, skin folds, feet, legs, thighs, tufts)	○	○	○	○	○
Strength assessment/postural control (core strength)	○	○	○	○	○

19. Gait assessment

Based on the responses, consensus was achieved on the following themes (70% of people identified the following assessments when describing the aspects of the lower limb visualised during a gait assessment. Therefore the following gait observations that are to be incorporated into the GALLOP are:

1. Shoulder symmetry (transverse, frontal plane rotation)
2. Arm swing (symmetry, guard position, flapping/flailing)
3. Hip (rotation, frontal plane motion, flexion, hip drop/riase)
4. Knee position (patella, flexion/extension/hyperextension, internal, frontal, external, genu varum/valgum, Q angle)
5. Heel contact (initial contact, motion, timing, heel left, rearfoot position)
6. Mid-stance (mid-foot position)
7. Toe off (Forefoot position, propulsion, symmetry, duration)
8. Other gait observations (Trendelenberg, limp, circumduction, abductory twist etc)

Preferred method of measure: Eyeball/Visual

The following assessments were also identified however consensus was not achieved. Please rate on the following scale your opinion of inclusion of the following gait observations

	Strongly Disagree	Disagree	Neutral	Agree	Strongly Agree
Head and neck position	○	○	○	○	○
Trunk/torso position and/or alignment	○	○	○	○	○
Length of stride	○	○	○	○	○
Angle of gait (foot progression angle)	○	○	○	○	○
Base of gait	○	○	○	○	○

Any comments about any of the above?

Thank you for taking the time to complete this survey. The final round will be emailed to you as soon as possible.
By clicking out of this survey you will not be able to re-enter and your answers will be saved.

If you would like to modify your responses, please use the back button or close and re-enter at a later date. Please remember you only have 2 weeks from this time to complete the survey. Your responses though will not be recorded until you click the button at the end of this question.
