# Supplementary material for: Gait and Lower Limb Observation of Paediatrics (GALLOP): development of a consensus based paediatric podiatry and physiotherapy standardised recording proforma
Source: J Foot Ankle Res. 2016 Mar 3;9:8. doi: 10.1186/s13047-016-0139-4 (PMC4778335; doi:10.1186/s13047-016-0139-4)
Supplement: Additional file 3: — GALLOP- Round 3. (PDF 251 kb) [file 13047_2016_139_MOESM3_ESM.pdf]

Supplementary File 3 - GALLOP – Round three.

University of
South Australia

GALLOP

Thank you for participating in this research.

This project aims to develop a **gait and lower limb assessment tool** that can be used to gain consistency in the type of questioning and assessment that is used with children during an initial assessment (or screen). Many comments have been made about some tests being used based on responses. The primary aim of the GALLOP is to guide the generalist through the minimal or required assessment of a paediatric gait and lower limb concern. We recognise other tests may be included if a negative result is obtained. As the expert panel, your opinion is important on if these additional tests should be included on this assessment.

To do this we are gathering your expertise and agreement during 3-4 rounds of questions therefore we are almost there!

To assist you in your responses thus far, we have also emailed you your responses from round 2.

Your responses are confidential and will only be known by the research team.

The survey should take under 10 minutes to complete.
You will have the chance at the end to make any comments if you wish.

If you have any questions please contact Simone Cranage (scranage@phcn.vic,gov.au) Cylie Williams (cyliewilliams@phcn.vic.gov.au) or Helen Banwell (HelenBanwell@unisa.edu.au)

For the following questions, we have provided the statement where 70% of consensus (**Round 1**) and agreement (**Round 2**) was achieved. During round 2, there were themes where less than 50% of participants agreed and these have been deleted and not displayed within this round (**Round 3**).

Where a theme had 51-69% of agreement, we ask you to again read the statements that have been produced through the first two rounds and then respond with your agreement of whether the theme of question or measure should be included.

Q1. What is your participant number?

1. Pre and post natal history
Based on the responses, consensus and agreement was achieved on the following themes (70% of people identified these themes).
Therefore the pre and post natal history questions that the parent/caregiver should be asked is:

1. Were there any complications during pregnancy? (Prompts: Health of the mother, use of medications)
Yes No
List:____
2. What was the term of the pregnancy? _________wks?
3. How was the baby delivered Vaginal Caesarean
4. Any any post-natal complications during or post delivery (Prompts: Resuscitations, NICU, Special Care
Nursery, congential abnormalities, medications, general health)
Yes No

List:____
5. Was the baby in a breech position? Yes No
6. What was the birth weight ____________
7. Was assistance required during labour Yes (forceps/ventouse) No
8. What was the APGAR score at: 1 minute ________ 5 minutes __________
9. Where any other medical professionals involved in the care of the baby at birth or within the first 14 days?

Yes (Birth Within 14 days) No
10. Is there a family history of foot or leg problems
Yes No

List:____

The following theme achieved agreement of 51-69% of participants. Please rate on the following scale your opinion of whether this theme should be included considering the current themes that consensus and agreement have been achieved on:

	Strongly Disagree	Disagree	Neutral	Agree	Strongly Agree
Labour	○	○	○	○	○

2. Developmental milestones/history

Based on the responses, consensus and agreement was achieved on the following themes (70% of people identified these themes).
Therefore the proposed developmental milestones/history questions that the parent/caregiver should be asked are:

Age of Sitting ______months
Age of Crawling _________________________months   Type of crawl__________________________
Age of Walking _____________months   Running ________________________years
Age of beginning to Jump ______________

The following themes achieved agreement of 51-69% of participants. Please rate on the following scale your opinion of whether these themes should be included considering the current themes that consensus and agreement have been achieved on:

	Strongly Disagree	Disagree	Neutral	Agree	Strongly Agree
Age of pull to stand	○	○	○	○	○
Age of beginning to cruise	○	○	○	○	○
Age of beginning to hop	○	○	○	○	○
Age of walking up/down stairs	○	○	○	○	○
Age of beginning to kick a ball	○	○	○	○	○

3. Other history questions

No consensus items were achieved in Round 1. Agreement was achieved (70% of participants agreed) in Round 2 on the following themes. Therefore the proposed additional history questions that the parent caregiver should be asked are:

1. What is the medical history of the child ______________________
2. Has there been any previous treatment of the feet or lower limbs (Prompt: interventions, orthotics, AFO's)
   Yes   No
   List: _____________________________________

3. Is there pain associated with the complaint (Prompt: Faces Pain Scale)
   Yes No
   List: ____________________________________

4. What is the typical footwear worn by the child ____________________________________

5. What is the child's recreational/sport activities or social activities ___________________

6. Has there be any observed sensory concerns (Prompt: surfaces/textures) ______________

7. What is the parental goals of assessment and/or treatment _________________________

8. What is the child's primary sitting position at play__________________________________

9. What is the child's primary sleeping position______________________________________

The following theme achieved agreement of 51-69% of participants. Please rate on the following scale your opinion of whether this theme should be included considering the current themes that consensus and agreement has been achieved on:

	Strongly Disagree	Disagree	Neutral	Agree	Strongly Agree
Height/weight	○	○	○	○	○

4. Hip Assessment

Based on the responses, consensus was achieved on the following themes (70% of people identified the following assessments used when assessing hip range of movement and the preferred measurement method). Therefore the following hip assessments to be incorporated into the GALLOP assessment are:

Internal/external rotation (knee flexed and/or extended) Left_________ Right___________

Thomas test (modified thomas test) Left________________ Right __________________

Hip abduction Left________________ Right __________________

Hip adduction Left _______________ Right ___________________

Preferred measurement methods: Goniometer

The following theme achieved agreement of 51-69% of participants. Please rate on the following scale your opinion of whether this theme should be included considering the current themes that consensus has been achieved on:

	Strongly Disagree	Disagree	Neutral	Agree	Strongly Agree
Quad ROM/tightness	○	○	○	○	○

5. Hamstring Assessment

Based on the responses, consensus was achieved on the following themes (70% of people identified the following assessments used when assessing hip range of movement and the preferred measurement method). Therefore the following hip assessment and method of measurement to be incorporated into the GALLOP assessment is:

Popliteal angle: Left _______________ Right ________________
Method: Angle finder

No further responses are required

6. Leg Length Assessment

No consensus was achieved in Round 1, however agreement was achieved (70% of participants agreed) in Round 2 on the following statement

1. Observation (frontal plane pelvic/shoulder tilt, scoliosis check, knee creases, head tilt, foot posture, gait)

The following themes achieved agreement of 51-69% of participants. Please rate on the following scale your opinion of whether these themes should be included considering the current themes that consensus and agreement have been achieved on:

	Strongly Disagree	Disagree	Neutral	Agree	Strongly Agree
ASIS-MM (Supine or standing)	○	○	○	○	○
Galleazi	○	○	○	○	○

7. Leg Length method of measurement

Based on the previous responses, no consensus was achieved in Round 1, however agreement was achieved (70% of participants agreed) in Round two on the following methods of measurement.

1.Tape measure
2. Palpation
3. Eyeball

No further responses are required

8. Rotational Profile

Based on the responses, consensus and agreement was achieved on the following themes (70% of people identified the following methods and tools when assessing rotational profile of the upper and lower leg). Therefore the following assessments to be incorporated into the GALLOP are:

1. Internal Hip ROM (hip flexed/extended, knee flexed/extended) Left___________ Right___________
2. External Hip ROM (hip flexed/extended, knee flexed/extended) Left ___________ Right ___________
3. Thigh foot angle (tibial torsion) Left _______________ Right __________________
4. Other (lumbar spine curve, shoulder symmetry, ASIS, knee position, metatarsus adductus)

The following themes achieved agreement of 51-69% of participants. Please rate on the following scale your opinion of whether these themes should be included considering the current themes that consensus and agreement have been achieved on:

	Strongly Disagree	Disagree	Neutral	Agree	Strongly Agree
Ryder's Test	○	○	○	○	○
Genicular rotation	○	○	○	○	○
Trans-malleolar angle/malleolar position	○	○	○	○	○

9. Rotational profile method of measurement.

Based on the responses, no consensus was achieved in Round 1, however agreement was achieved (70% of particpants agreed) in Round 2 on the following method of measurement.

1. Goniometer

The following method of measurement achieved agreement of 51-69% of participants. Please rate on the following scale your opinion of whether this method should be included considering the current method that agreement has been achieved on:

	Strongly Disagree	Disagree	Neutral	Agree	Strongly Agree
Eyeball	◯	◯	◯	◯	◯

10. Ankle range of movement

Based on the responses, consensus and agreement was achieved on the following themes (70% of people identified the following assessments used when assessing ankle range of movement). Therefore the following assessments to be incorporated into the GALLOP are:

1. Non weight-bearing ankle dorsiflexion (knee extended): Left __________ Right ___________
2. Non weight-bearing ankle dorsiflexion (knee flexed): Left __________ Right ____________
3. Weight-bearing lunge test (knee flexed, knee extended) Left _________ Right ____________
4. Able to squat Yes_______ No ________ N/A
5. Able to walk on toes Yes_______ No ________ N/A
6. Able to heel walk Yes_______ No ________ N/A

No further responses are required

11. Ankle range of movement method of measurement

Based on the responses, no consensus was achieved in Round 1, however agreement was achieved (70% of participants agreed) in Round two on the following method:

1. Goniometer

The following methods of measurement achieved agreement of 51-69% of participants. Please rate on the following scale your opinion of whether these should be included considering the current method that agreement has been achieved on:

	Strongly Disagree	Disagree	Neutral	Agree	Strongly Agree
Digital inclinomter	◯	◯	◯	◯	◯
Eyeball measurement	◯	◯	◯	◯	◯

12. Presence of genu varum/genu valgum

Based on the responses, consensus and agreement was achieved on the following themes (70% of people identified the following assessments used measuring the presence of genu varum/genu valgum. Therefore the following assessments to be incorporated into the GALLOP are:

1. Intercondylar distance (WB) ________cm
2. Intermalleolar distance (WB) ________ cm

Method of measurement
1. Use of a tape measure/ruler

The following theme achieved agreement of 51-69% of participants. Please rate on the following scale your opinion of whether these themes should be included considering the current themes that consensus and agreement have been achieved on:

	Strongly Disagree	Disagree	Neutral	Agree	Strongly Agree
Intercondylar distance (non-WB)	○	○	○	○	○
Intermalleolar distance (non-WB)	○	○	○	○	○

13. Foot Posture

Based on the responses, consensus and agreement was achieved on the following themes (70% of people identified the following assessments used when assessing foot posture). Therefore the following assessments to be incorporated into the GALLOP are:

1. FPI-6 Left ___________ Right _____________

2. Hubscher maneuver (Jack's test)
Left Positive/Negative Right Positive/Negative

3. Subtalar joint axis
Left Medial/Typical/Lateral Right Medial/Typical/Lateral

4. Subtalar joint ROM
Left Restricted/Typical/Hypermobile Right Restricted/Typical/Hypermobile

5. Forefoot to rearfoot relationship
____

6. Midtarsal joint non weightbearing ROM
Left Restricted/Typical/Hypermobile Right Restricted/Typical/Hypermobile

5. 1st MPJ Non weightbearing ROM
Left Restricted/Typical/Hypermobile Right Restricted/Typical/Hypermobile

6.. Ability to stand on tip toes
Left Yes/No/NA Right Yes/No/NA

7. Does the rearfoot resupinate while on tiptoes
Left Yes/No/N/A Right Yes/No/N/A

Method of measurement: Eyeball

The following themes achieved agreement of 51-69% of participants. Please rate on the following scale your opinion of whether these themes should be included considering the current themes that consensus and agreement have been achieved on:

	Strongly Disagree	Disagree	Neutral	Agree	Strongly Agree
Resting Calcaneal Stance Position	○	○	○	○	○
Supination resistance	○	○	○	○	○

14. Gross motor ability/balance

Based on the responses, consensus and agreement was achieved on the following themes (70% of people identified the following assessments used when measuring gross motor ability and/or balance). Therefore the following assessments to be incorporated into the GALLOP are:

Observations of achievement:

1. Running Yes/No/N/A Observation: ______________
2. Jumping Yes/No/N/A Observation: ______________
3. Skipping Yes/No/N/A Observation: ______________
4. Hopping Yes/No/N/A Observation: ______________
5. Single leg stance (eyes open, timed) Left _________ Right _____________
6. Single leg stance (eyes closed, timed) Left _________ Right ____________
7. Ability to go up/down stairs Yes/No/N/A Observation: ______________
8. Other functional tasks (throwing, catching, kicking a ball, animal walks, sport specific activities)
12. Other observations (fatigue, agility, maturity of pattern, coordination of movements, symmetry, strength)
13. Use of standardised assessment (Himat, BOT-2, Berg balance scale, AIMS, Peabody, ASQ etc.
14. Presence of reciprocal arm swing Yes/No/N/A Observation: ______________
15. Quality of body movement (symmetry, coordination) Observation: ______________

The following themes achieved agreement of 51-69% of participants. Please rate on the following scale your opinion of whether these themes should be included considering the current themes that consensus and agreement have been achieved on:

	Strongly Disagree	Disagree	Neutral	Agree	Strongly Agree
Climbing ability	○	○	○	○	○
Balance beam	○	○	○	○	○

15. Reflexes

Based on the responses, consensus was achieved on the following themes (70% of people identified the following reflexes tested in the lower limb. Therefore the reflex tests to be incorporated into the GALLOP are:

1. Patella (knee jerk, quadriceps) reflex
2. Achilles (ankle jerk, gastrocnemius) reflex
3. Plantar reflex (up or down going)

No further responses are required

---

16. Based on the responses, consensus was achieved (70% of people identified the following preferred grading method of responses) on the following scale:

0: Absent reflex
1+: trace, or seen only with reinforcement
2+: normal
3+: brisk
4+: nonsustained clonus (i.e., repetitive vibratory movements)
5+ sustained clonus

No further responses are required.

17. Neurological other

Based on the responses, no consensus was achieved in Round 1, however agreement was achieved (70% of participants agreed) in round Two on the following themes. Therefore the follow assessments to be included are:

1. Muscle tone (passive, active, spasticity, rigidity)
2. Presence of a catch (R1/R2) Site: ___________________________
3. Presence of a clonus Site:_________________________________
4. Gower's sign Yes/No
5. Dorsiflexion strength (Grading 0-5) Left   Right
6. Plantarflexion strength (Grading 0-5) Left   Right
7. Inversion strength (Grading 0-5) Left   Right
8. Eversion strength (Grading 0-5) Left   Right

The following theme achieved agreement of 51-69% of participants. Please rate on the following scale your opinion of whether this theme should be included considering the current themes that agreementhas been achieved on:

	Strongly Disagree	Disagree	Neutral	Agree	Strongly Agree
Sensory assessment (perception, discrimination, sensation, proprioception, monofilament)	○	○	○	○	○

18. Assessment other

Based on the responses, no consensus was achieved in Round 1, however agreement was achieved (70% of participants agreed) in round 2 on the following assessments:

1. Beighton score __________
2. Other observations (syndactlyl, skin folds, feet, legs, thighs, tufts) ___________________

The following assessment achieve agreement of 51-69% of participants. Please rate on the following scale your opinion of whether this assessment should be included:

	Strongly Disagree	Disagree	Neutral	Agree	Strongly Agree
Toe walking tool	○	○	○	○	○

19. Gait assessment

Based on the responses, consensus and agreement was achieved on the following themes (70% of people identified the following assessments when describing the aspects of the lower limb visualised during gait assessment). Therefore the following gait observations be incorporated into the GALLOP are:

1. Shoulder symmetry (transverse, frontal plane rotation)
2. Arm swing (symmetry, guard position, flapping/flailing)
3. Hip (rotation, frontal plane motion, flexion, hip drop/rise)
4. Knee position (patella, flexion/extension/hyperextension, internal, frontal, external, genu varum/valgum)
5. Heel contact (initial contact, motion, timing, heel lift, rearfoot position)
6. Mid-stance (mid-foot position)
7. Toe off (Forefoot position, propulsion, symmetry, duration)
8. Other gait observations (Trendelenberg, limp, limb circumduction, abductory twist etc)

9. Head and neck position
10. Trunk/torso position and/or alignment
11. Angle of gait (foot progression angle)
12. Base of gait

Preferred method of measure: Eyeball/Visual

The following theme achieved agreement of 51-69% of participants. Please rate on the following scale your opinion of whether this theme should be included considering the current themes that agreement has been achieved on:

	Strongly Disagree	Disagree	Neutral	Agree	Strongly Agree
Length of stride	○	○	○	○	○

Any comments about any of the above?

Thank you for taking the time to complete this survey. The final round will be emailed to you as soon as possible. Within the final round where there has greater than 70% agreement or less than 50% agreement, a final copy of the statements will be emailed only and the rounds ceased. Only if there is outstanding themes of 51-69% will these be circulated for one final round.

By clicking out of this survey you will not be able to re-enter and your answers will be saved.

If you would like to modify your responses, please use the back button or close and re-enter at a later date. Please remember you only have 2 weeks from this time to complete the survey. Your responses though will not be recorded until you click the button at the end of this question.
