# Supplementary material for: Gait and Lower Limb Observation of Paediatrics (GALLOP): development of a consensus based paediatric podiatry and physiotherapy standardised recording proforma
Source: J Foot Ankle Res. 2016 Mar 3;9:8. doi: 10.1186/s13047-016-0139-4 (PMC4778335; doi:10.1186/s13047-016-0139-4)
Supplement: Additional file 4: — GALLOP recording proforma. (DOCX 118 kb) [file 13047_2016_139_MOESM4_ESM.docx]

GALLOP 1 of 2

Name_____________________________ Date of Birth___/__/___ Gender: Male/Female

***Postnatal History (Complete or circle)***

| Gestation:___________weeks | Birth weight:________grams | APGAR 1 min____5min____ |
| --- | --- | --- |
| Vaginal birth:  Spontaneous/Induced | Instrumentation at birth  Forceps/Ventouse | Caesarean: Emergency/Planned  Reason:____________________ |
| Breech: Yes/No  Complications_________________________________________  _____________________________________________________ | | Other health professionals involved at birth or in first 14 days:_______________________ |

***Age of skill acquisition (record in months)***

| Sitting: ___________ | Crawling:__________ | Crawl type:__________ |
| --- | --- | --- |
| Walking: _________ | Running:__________ | Jumping:____________ |
| ***History:*** |  |  |
| Medical/Family_______________  ___________________________ | Previous treatment:______  _______________________ | Previous Pain:____________  _________________________ |
| Footwear:___________________  ___________________________ | Sport:__________________  _______________________ | Sensory concerns:_________  ________________________ |
| Weight: ______kg | Height: ______cm | BMI: |

***Observation of ability to perform the following appropriate to age (Circle)***

| Squatting: Yes/No/NA | Running: Yes/No/NA | Jumping: Yes/No/N/A |
| --- | --- | --- |
| Skipping: Yes/No/NA | Hopping: Yes/No/NA | Single Leg Stance  Left: Yes/No/N/A  Right: Yes/No/N/A |
| Ability to go up/down stairs:  Yes/No/NA | Observation of functional tasks:  __________________________ | Quality of body movement:  __________________________ |

***Other Observations***

***______________________________________________________________________________________________________________________________________________***

GALLOP 2 of 2

***Pain***


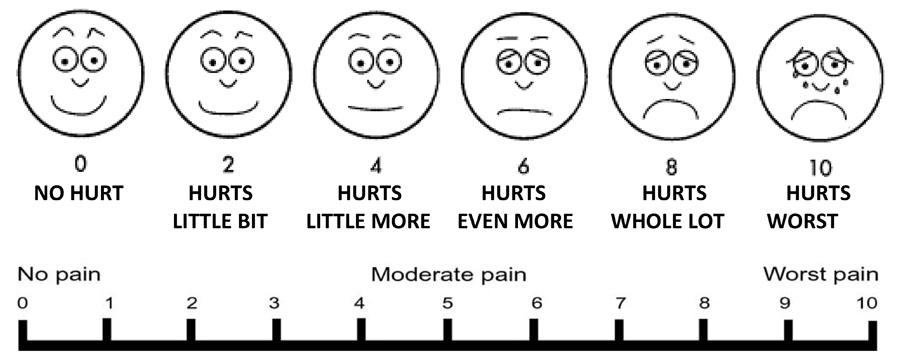


***Biomechanical Assessments:***

|  | **Left** | **Right** |  | **Left** | **Right** |
| --- | --- | --- | --- | --- | --- |
| Hip: Internal ROM |  |  | Hip External ROM |  |  |
| Modified Thomas Test |  |  | Hip abduction |  |  |
| Popliteal angle |  |  | Foot thigh angle |  |  |
| Ankle WBL/NWB Straight |  |  | Ankle WBL/NWB Bent |  |  |
| Foot Posture Index-6 |  |  | Beighton score _______/9 |  |  |

| Inter-condylar distance:  _________cm  Inter-malleoli distance:  _________cm | *Limb Length Discrepancy**:  Left=Right  Left>Right______cm  Left<Right______cm | Other observations of rotation, limb length:_______________  _________________________ |
| --- | --- | --- |

***Neurology:***

|  | Left | Right |  | Left | Right |
| --- | --- | --- | --- | --- | --- |
| Patella Reflex (0-4) |  |  | Achilles Reflex (0-4) |  |  |
| Plantar Reflex (Up/down) |  |  | Ankle Catch (Yes/No) |  |  |
| Ankle Clonus (Yes/No) |  |  | Gower’s Sign |  |  |
| Dorsiflexion strength (0-5) |  |  | Plantarflexion Strength (0-5) |  |  |
| Inversion strength (0-5) |  |  | Eversion strength (0-5) |  |  |
| Observation of muscle tone or neurological signs: | | |  |  |  |

***Gait****

|  | Left | Right |  | Left | Right |
| --- | --- | --- | --- | --- | --- |
| Head and neck position |  |  | Trunk/torso |  |  |
| Arm swing |  |  | Hip |  |  |
| Knee |  |  | Heel contact |  |  |
| Midstance |  |  | Toe-off |  |  |
| Angle of gait |  |  | Base of gait |  |  |
| Other gait comments: |  |  |  |  |  |
|  |  |  |  |  |  |

GALLOP (Addendum)

***Tips for completion of free text questions instructions for podiatrists and physiotherapists:***

***Ankle range of motion***

The weightbearing lunge (WBL) should be performed if the child is able to put their heel to the group due to age specific normative values and higher reliability than the non weight bearing test (NWB)

***Observation of functional tasks:***

Is the child able to perform activities appropriate to their age such as: throwing a ball, catching a ball, kicking a ball, animal walks, sport specific activities

***Quality of movement:***

Does the child perform tasks symmetrically or with smooth movement? Is their movement clumsy, jerky or asymmetrical?

***Other observations of rotation, limb length****:

Presence of metatarsus adductus graded by severity and flexibility, uneven creases behind the knees or buttocks.

***Gait Observations****

Head and neck position: Tilt or lean

Trunk or torso position: Lordosis, kyphosis, tilt or lean

Arm swing: symmetry, guard position, flapping/flailing

Hip: rotation, frontal plane motion, flexion, hip drop or raise

Knee position: patella position, flexion, extension, hyperextension

Heel contact: initial contact, motion, timing, lift or rear foot position

Mid-stance: midfoot position

Toe – off: forefoot position, propulsion, symmetry, duration

Foot progression angle: Appropriate for age, too many toes

Base of gait: Narrow, scissor, wide

Other gait comments: Trendelenberg, limp, circumduction, abductory twist

*** Indicate items without paediatric age-specific normative values or low reliability therefore clinicians should use and interpret with caution**
